# LabPipe: an extensible bioinformatics toolkit to manage experimental data and metadata

**DOI:** 10.1186/s12859-020-03908-5

**Published:** 2020-12-02

**Authors:** Bo Zhao, Luke Bryant, Rebecca Cordell, Michael Wilde, Dahlia Salman, Dorota Ruszkiewicz, Wadah Ibrahim, Amisha Singapuri, Tim Coats, Erol Gaillard, Caroline Beardsmore, Toru Suzuki, Leong Ng, Neil Greening, Paul Thomas, Paul Monks, Christopher Brightling, Salman Siddiqui, Robert C. Free

**Affiliations:** 1 Department of Respiratory Sciences, University of Leicester, Leicester, UK; 2 NIHR Leicester Biomedical Research Centre, University of Leicester, Leicester, UK; 3 Department of Chemistry, University of Leicester, Leicester, UK; 4 Department of Cardiovascular Sciences, Cardiovascular Research Centre, University of Leicester, Leicester, UK; 5 Department of Chemistry, Loughborough University, Loughborough, UK

**Keywords:** Metadata, Data management, Biomedical, Breathomics

## Abstract

**Background:**

Data handling in clinical bioinformatics is often inadequate. No freely available tools provide straightforward approaches for consistent, flexible metadata collection and linkage of related experimental data generated locally by vendor software.

**Results:**

To address this problem, we created LabPipe, a flexible toolkit which is driven through a local client that runs alongside vendor software and connects to a light-weight server. The toolkit allows re-usable configurations to be defined for experiment metadata and local data collection, and handles metadata entry and linkage of data. LabPipe was piloted in a multi-site clinical breathomics study.

**Conclusions:**

LabPipe provided a consistent, controlled approach for handling metadata and experimental data collection, collation and linkage in the exemplar study and was flexible enough to deal effectively with different data handling challenges.

## Background

A key challenge in clinical bioinformatics is handling the collation and collection of experimental data sets from multiple sites and research groups. While some vendor software is fully automated and provides an end-to-end system for collecting data and metadata, in many cases this is not the case and software is closed, proprietary and limited with a lack of external connectivity. This means data management is often a manual ad-hoc process, which leads to an approach that is inadequate, slow and potentially error-ridden [1].

While there are open source tools available which improve on this situation [2, 3], our team required a tool which was flexible enough to be able to handle multiple different configurations for metadata entry and experimental data linkage depending on the data being collected and the vendor software being used on local PCs.

In response, we developed a new bioinformatics toolkit (LabPipe) which enabled us to create customised re-usable configurations for consistent experimental metadata and data management. In this article, the toolkit is described, along with how it was deployed in an exemplar study, handling breathomics data collected across multiple sites and analytical chemistry platforms.

## Implementation

**Fig. 1:**
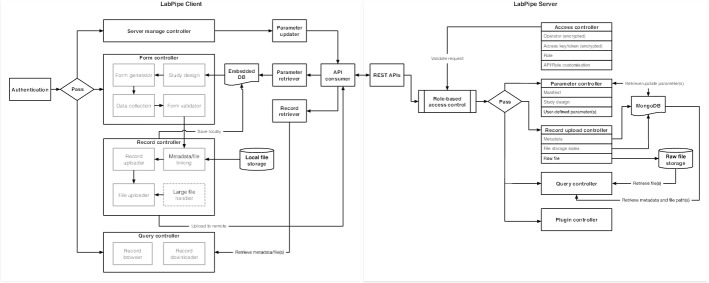
The technical architecture of LabPipe. Controllers in LPS and LPC handle specific aspects of the system. Communication between the two is made through the LPS REST API. Metadata and data files are stored locally in an embedded database and local file storage respectively, and uploaded if or when a network connection becomes available. Additional details are provided in the main text

LabPipe uses a modular client–server architecture (Fig. 1) with an extensible plugin mechanism for adding new features. The toolkit consists of LabPipe Server (LPS): a group of light-weight REpresentational State Transfer (REST) based APIs; and LabPipe Client (LPC): a locally installed front-end to support and manage local data/meta-data collection and configuration. The toolkit was created iteratively with Continuous Integration approaches, and includes unit tests covering all data operations within the code-base.

The toolkit was developed taking into account the STRIDE threat model [4] and as such includes a number of security measures. At a basic level it is designed to prevent attacks through potential vectors (e.g. XSS and SQL injection). Additionally, user credentials are encrypted in both LPC and LPS to minimise the risk of identify theft. Enforcing default HTTPS connections also prevents man-in-the-middle attacks. Furthermore, the LPS APIs are protected from external injection by sanitising incoming data and rejecting unauthorised query operators such as $where and both LPC and LPS maintain logs when there is a change to configuration or record data, so if there are unauthorised changes it can be traced. Other access limits include specific access controls on the role-based API to restrict user access within allocated boundaries and a customisable rate limit on each API to prevent basic denial-of-service attacks. Despite the inclusion of these security measures, we would not advise that LabPipe is used to collect identifiable or sensitive information without additional mitigation and testing.

The APIs provided by LPS handle configuration, data and metadata download/upload and are protected by role-based authorisation. The server stores configurations in a document-based NoSQL database, which allows more flexibility than a relational database. These configurations include pre-defined common parameters such as data collection form templates and study design components (location, operator, instrument). LPS also supports user-defined parameters. Both pre-defined and user-defined parameters are grouped into collections. An editable manifest tells the LPC instances which parameters to retrieve. Access control parameters such as role, access token and API-role mapping are also part of the server configurations. When networked, these configurations are fetched by local LPCs upon set up/use and are also cached locally to enable offline use. It should be possible to setup an LPS instance on any server with Java version 8 or higher and MongoDB version 4 or higher installed, including cloud-based services such as Amazon Web Services. Raw data files are stored as is on the server, with a link between the metadata and the data file stored in MongoDB. Larger data files (defaults to a minimum of 50 MB), are transferred in pieces and re-assembled at the end using a checksum to ensure integrity. LPS uses a combination of security token and user/password based approaches to handle authentication.

LPC was built using the Electron framework and acts as both a data collection assistant and a configuration manager for an LPS instance. In the former role, LPC retrieves configurations and form templates from a connected LPS and generates forms for defined processes. In the latter role, users allocated with appropriate privileges can remotely view, add and edit configurations and form templates on the connected LPS, which can be customised to cover different aspects of metadata collection (e.g. from sample collection quality control checks to clinical observations). Furthermore, field validation can be added (e.g. minimum; maximum; specific data types; regular expressions; basic ontology support) to provide data limits and appropriate errors and warnings to the user. Basic ontology support is setup through an optional field property which specifies which ontology should be used and the ID of the concept. The LPS instance verifies the ontology using the BioPortal API [5]. LabPipe also provides support for different locales, so that it can be used between countries with different numerical and date/time criteria.

A step-by-step guide to setup and use LabPipe is available as Additional file 1 and at docs.labpipe.org/step-by-step. In brief, it involves setting up a central LPS instance and installing the LPC tool on each of the vendor software PCs, then configuring them to connect to the LPS instance. Following this, a user with the superuser role configures the connected LPS with appropriate forms/fields. Once configuration is complete, LabPipe can be used to manage the data and metadata collection process. Users enter metadata into forms generated by the LPC (according to the LPS configuration) at the point of vendor software data generation/collection. The local LPC then links this metadata to the data files generated by the vendor software/guides the user to do this, and uploads them to the LPS instance.

For testing purposes, an LPS instance has been made available at https://try-server.labpipe.org. But an LPC will need to be installed on a local PC in order to test it. Some example form/field configurations are also provided which can be implemented.

## Results and discussion

**Fig. 2:**
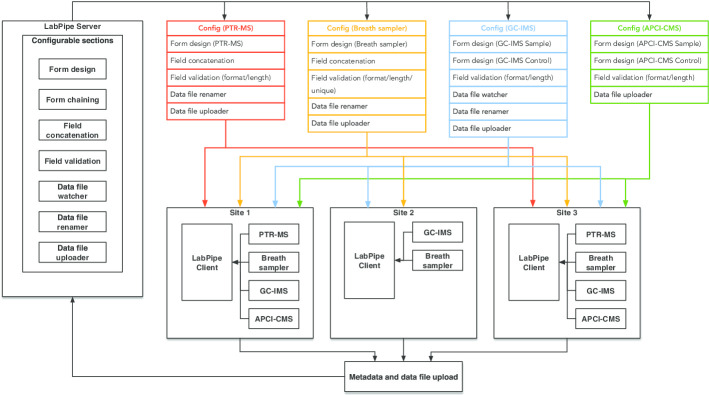
LabPipe setup and workflow in the exemplar EMBER breathomics study. The EMBER LPS was setup to handle standard operating procedures for four analytical chemistry instrument data/sample collection techniques including: proton-transfer-reaction mass spectrometry (PTR-MS); gas chromatography ion mobility spectrometry (GC-IMS); atmospheric pressure chemical ionisation compact mass spectrometry (APCI-CMS) and a breath sampler device which collected samples for later analysis. Re-usable configurations for each technique (shown in the diagram with distinct colours) were setup with data collection forms containing appropriate field processing/validations and data file handlers. Data collection at each site was managed through LPC instances which loaded appropriate configurations to generate metadata forms and guide the user through any manual steps required when saving data files. Once these steps were completed, the LPC automatically uploaded data and metadata to the LPS.

LabPipe was piloted in the EMBER study [6], a breathomics study involving staff from different backgrounds with various skill sets. Figure 2 shows how LabPipe was setup to support metadata collection and sample data acquisition from 10 analytical chemistry instruments, covering four different techniques for breath sampling across three sites.

Breath sampling instruments in the study included both online technologies (in which samples were analysed in real-time on the instrument) and offline technologies (in which samples were collected and later transferred to be analysed in the lab), and LabPipe was able to handle data management for these different scenarios. Each patient visit involved the generation of multiple sets of data using vendor software, which was supported through LabPipe and linked to metadata entered at the time samples/sample data were acquired.

LabPipe was enhanced through an interdisciplinary collaboration between informaticians, researchers and clinical staff. The aim was to create a platform which was accessible and easy to use. This was helped through an iterative development process guided by qualitative surveys and informal team discussions to assess pain-points in the software. Members of our team agreed that the resulting wizard-based system was better than alternative approaches as it could be setup to guide non-technical members through entire standard operating procedures. In response to researcher’s needs, support was also added to LabPipe for controlled access to data through the LPS API and a search/data export user interface in LPC which made it easier for researchers to carry out their analyses.

The introduction of LabPipe into the EMBER study reduced manual data handling and management, saved time and increased efficiency. Prior to its deployment, paper-based records, manual transcription and removable storage devices were used to manage metadata and data from local PCs. By deploying LabPipe and a guided process for metadata and data collection/linkage, the likelihood of erroneous data and data loss was reduced.

Existing freely available bioinformatics tools with the same scope as LabPipe were investigated [2, 3] and only one comparable tool to LabPipe was identified: MASTR-MS.

As in LabPipe, the mechanism for vendor software data acquisition in MASTR-MS is a tool which runs in the background on the client machine (the DataSync Client). However, unlike LabPipe this runs as a light-weight service; and linked metadata is collected using the MASTR-MS web application. While this approach has some advantages, it means that MASTR-MS cannot handle intermittent/no network connections effectively when collecting both metadata and data.

Furthermore, while MASTR-MS provides some flexibility in terms of data collection, it is specifically tailored to metabolomics. In contrast, LabPipe metadata forms/fields can be customised to collect different types of experimental metadata and the vendor data collection/linkage approach can be adapted using the extensible architecture. For example, depending on the vendor tools used to collect data, linkage can be implemented by generating unique file identifiers (concatenating entered data fields into an identifier); through a file watcher/notifier; or by building a vendor software specific data linkage plugin. The extensibility of LabPipe also enables other types of features to be developed. For example, during the EMBER study, a plugin was created which would send researchers notification emails when breath sample metadata and/or data had been uploaded to LabPipe.

Critically, unlike LabPipe, which is being actively developed, it is unclear whether MASTR-MS is still being supported. The latest release was in 2017 and it was developed using a now end-of-life programming language (Python 2).

Although the exemplar presented here is a breathomics study, we believe that LabPipe is flexible enough to handle data collection from vendor software for other experiment types where a similar metadata/data management approach would be effective. This ability will be further enhanced by our plan to add support for data standards such as ISA [7] and mzTab [8]. To facilitate this, ontology support will be improved, and form handling capabilities will be extended to allow more complex metadata configurations, such as multi-faceted and embedded forms.

## Conclusion

Through its deployment in the exemplar study we have shown that LabPipe provides a consistent, controlled way to handle metadata and experimental data collection, collation and linkage for clinical bioinformatics. We believe it provides a straight forward, fully configurable and extensible approach to experiment data handling which could help address the needs of modern lab management.

## Availability and requirements

- Project name: LabPipe.
- Project home page: https://labpipe.org.
- Operating system(s): Platform independent.
- Programming language: Kotlin (Server), JavaScript (Client).
- Other requirements: Java 8 or higher, MongoDB 4 or higher, 200MB disk space, 500MB RAM (Server); 200MB disk space, 2GB RAM (Client).
- License: GNU General Public Licence v3.0 (GPL-3.0).
- Any restrictions to use by non-academics: None.

## Supplementary information

**Additional file 1:** A step-by-step guide to setting up LabPipe using an example configuration.

## Data Availability

The LabPipe source code is available at https://github.com/rcfgroup/labpipe.

## Abbreviations

LPS: LabPipe Server
LPC: LabPipe Client
EMBER: East Midlands Breathomics Pathology Node

## References

[1] D’Alessandro A, Giardina B, Federica G, Timperio AM, Zolla L (2012). Clinical metabolomics: the next stage of clinical biochemistry. Blood Transfus.

[2] Müller H, Malservet N, Quinlan P, Reihs R, Penicaud M, Chami A, Zatloukal K, Dagher G (2017). From the evaluation of existing solutions to an all-inclusive package for biobanks. Health Technol.

[3] LIMSWiki. https://www.limswiki.org. Accessed 27 Oct 2020.

[4] Kohnfelder L, Praerit G. The threats to our products. https://adam.shostack.org/microsoft/The-Threats-To-Our-Products.docx (1999). Accessed 27 Oct 2020.

[5] Whetzel PL, Noy NF, Shah NH, Alexander PR, Nyulas C, Tudorache T, Musen MA (2011). BioPortal: enhanced functionality via new Web services from the National Center for Biomedical Ontology to access and use ontologies in software applications. Nucleic Acids Res.

[6] Ibrahim W, Wilde M, Cordell R, Salman D, Ruszkiewicz D, Bryant L, Richardson M, Free RC, Zhao B, Yousuf A, White C, Russell R, Jones S, Patel B, Awal A, Phillips R, Fowkes G, McNally T, Foxon C, Bhatt H, Peltrini R, Singapuri A, Hargadon B, Suzuki T, Ng LL, Gaillard E, Beardsmore C, Ryanna K, Pandya H, Coates T, Monks PS, Greening N, Brightling CE, Thomas P, Siddiqui S (2019). Assessment of breath volatile organic compounds in acute cardiorespiratory breathlessness: a protocol describing a prospective real-world observational study. BMJ Open.

[7] Rocca-Serra P, Brandizi M, Maguire E, Sklyar N, Taylor C, Begley K, Field D, Harris S, Hide W, Hofmann O, Neumann S, Sterk P, Tong W, Sansone SA (2010). ISA software suite: supporting standards-compliant experimental annotation and enabling curation at the community level. Bioinformatics.

[8] Griss J, Jones AR, Sachsenberg T, Walzer M, Gatto L, Hartler J, Thallinger GG, Salek RM, Steinbeck C, Neuhauser N, Cox J, Neumann S, Fan J, Reisinger F, Xu Q-W, del Toro N, Pérez-Riverol Y, Ghali F, Bandeira N, Xenarios I, Kohlbacher O, Vizcaíno JA, Hermjakob H (2014). The mztab data exchange format: communicating mass-spectrometry-based proteomics and metabolomics experimental results to a wider audience. Mol Cell Proteomics.

