# Supplementary material for: LabPipe: an extensible bioinformatics toolkit to manage experimental data and metadata
Source: BMC Bioinformatics. 2020 Dec 2;21:556. doi: 10.1186/s12859-020-03908-5 (PMC7709404; doi:10.1186/s12859-020-03908-5)
Supplement: Supplementary file 1 — Additional file 1: A step-by-step guide to setting up LabPipe using an example configuration. [file 12859_2020_3908_MOESM1_ESM.pdf]

LabPipe step-by-step setup guide

Supplementary information for setting up a basic LabPipe Server/Client.

Install server prerequisites

LabPipe Server requires:

- MongoDB
  Tested with MongoDB 4.4.1 Community Edition and MongoDB Atlas 4.2.10 Enterprise
- Java
  Tested with OpenJDK 1.8.0.212 and OpenJDK 11.0.2

1. Follow the guide to install MongoDB
2. Follow the guide to install OpenJDK 8 or OpenJDK 11

Download LabPipe Server

Download the latest release (JAR file) at https://github.com/rcfgroup/labpipe-server-public/releases/latest.

Download LabPipe Client

Download the latest release at https://github.com/rcfgroup/labpipe-client-public/releases/latest based on your operating system:

- Windows
  - labpipe-client.Setup.[version].exe
    Click and run.
- Mac
  - labpipe-client-[version]-mac.zip
    Uncompress and move **labpipe-client.app** to **Applications**, then click and run. It may ask for permission to run and to access your disk.
- Linux
  - labpipe-client-[version].AppImage
    Make the downloaded file executable using `chmod a+x`, then use `./labpipe-client-[version].AppImage` to run.

Configure LabPipe Server

```
java -jar labpipe-server-v{x.x.x}.jar check
```

If you are using LabPipe Server for the first time, this will use built-in default configs to check whether LabPipe Server can work. A config file `config.toml` will also be generated. Here is an example of the file:

config.toml

```
[labpipe]
port = 4567          # The port LabPipe Server will be running on

[labpipe.database]
host = "localhost"  # MongoDB host
port = 27017        # MongoDB port, can leave as default if using srv mode
name = "labpipe"    # MongoDB database name
user = ""           # MongoDB username
password = ""       # MongoDB password
useSrv = false      # Use srv mode to connect

[labpipe.email]
host = "localhost"  # Email server host
port = 25           # Email server port
user = ""           # Email server username
password = ""       # Email server password
fromName = "LabPipe Notification"     # Sender name for notification email
fromAddress = "no-reply@labpipe.org"  # Sender email for notification email

[labpipe.parameter]
manifest = []       # Currently not in use

[labpipe.security]
enforceSsl = true       # Forward all HTTP connection to HTTPS
rateLimitPublic = 100   # Rate limit for public APIs, per minute

[labpipe.storage]
cache = "/path/to/labpipe"            # Cache directory
upload = "/path/to/labpipe/uploaded"  # Directory for uploaded file
parts = "/path/to/labpipe/parts"      # Directory for uploaded chuncked file parts

```

Once you have modified the `config.toml` to your working database and email server, again run

```
java -jar labpipe-server-v{x.x.x}.jar check
```

If everything works fine, you should see something like

```
[main] INFO uk.ac.le.ember.labpipe.server.controllers.EmailController -
    Email server connection successful.
[main] INFO uk.ac.le.ember.labpipe.server.controllers.DatabaseController -
    Database connection successful.
```

Now you can initialise the database with:

```
java -jar labpipe-server-v{x.x.x}.jar init
```

This will populate the database with three collections:

Collection	Description
ROLES	User or token roles for role-based access control
API_ACCESS_ROLES	Role-based control and rate limit for specific APIs
CLIENT_SETTINGS	A manifest tells LabPipe Client which parameter(s) to retrieve

Once `init` is completed, the contents for the three collections should look like

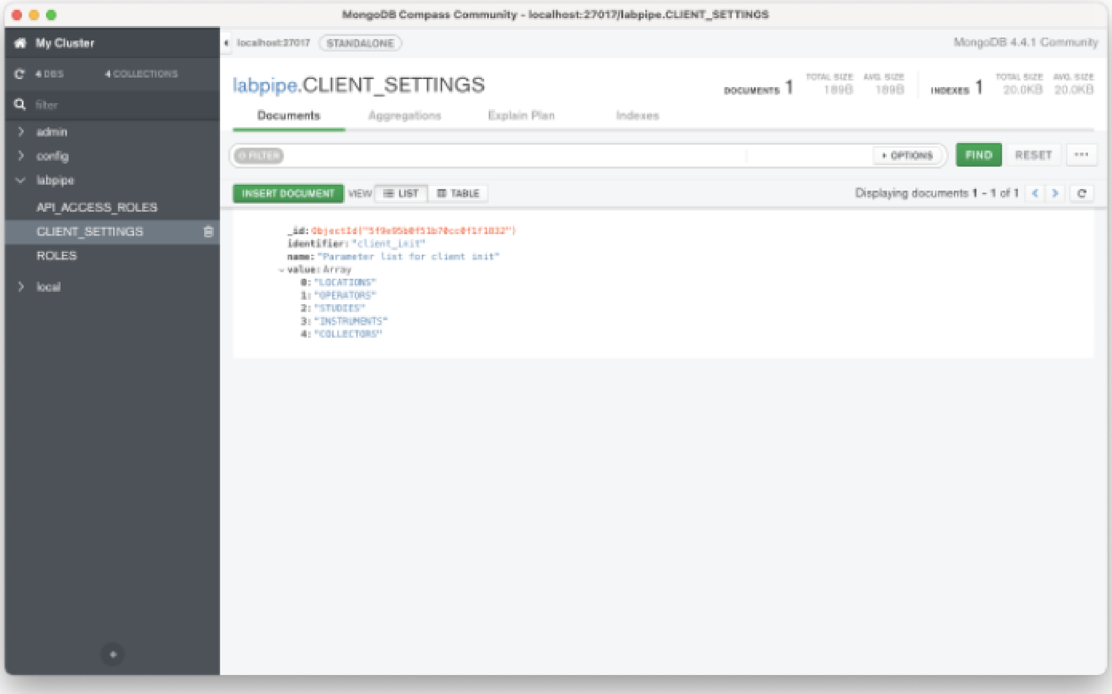

CLIENT_SETTINGS collection viewed using MongoDB Compass

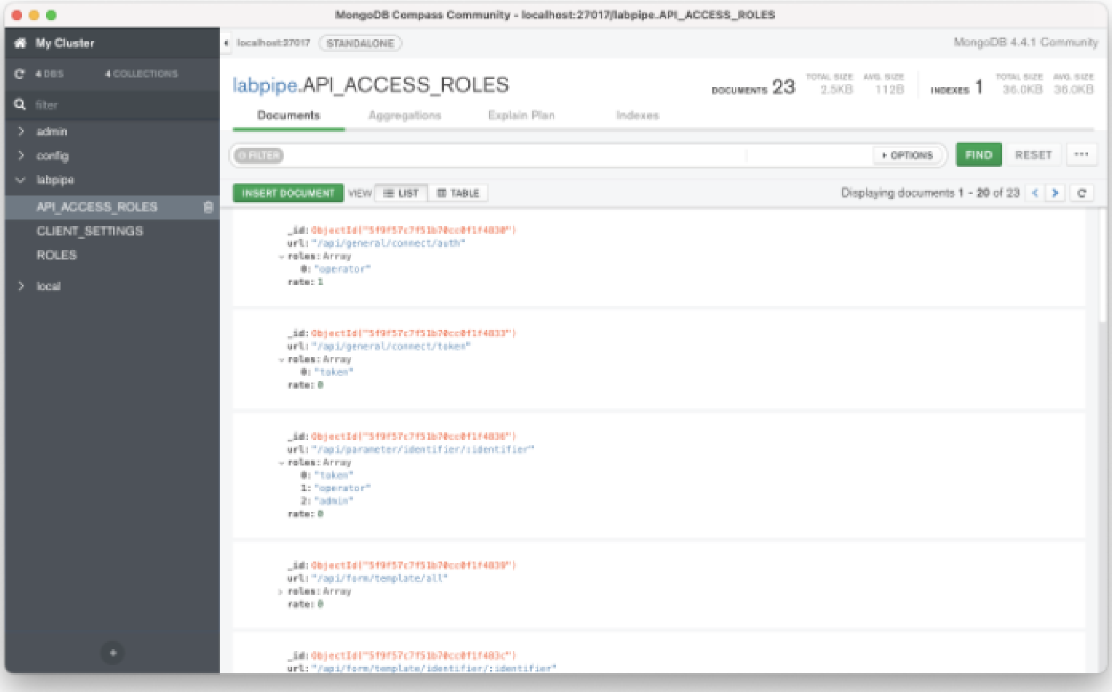

API_ACCESS_ROLES collection viewed using MongoDB Compass

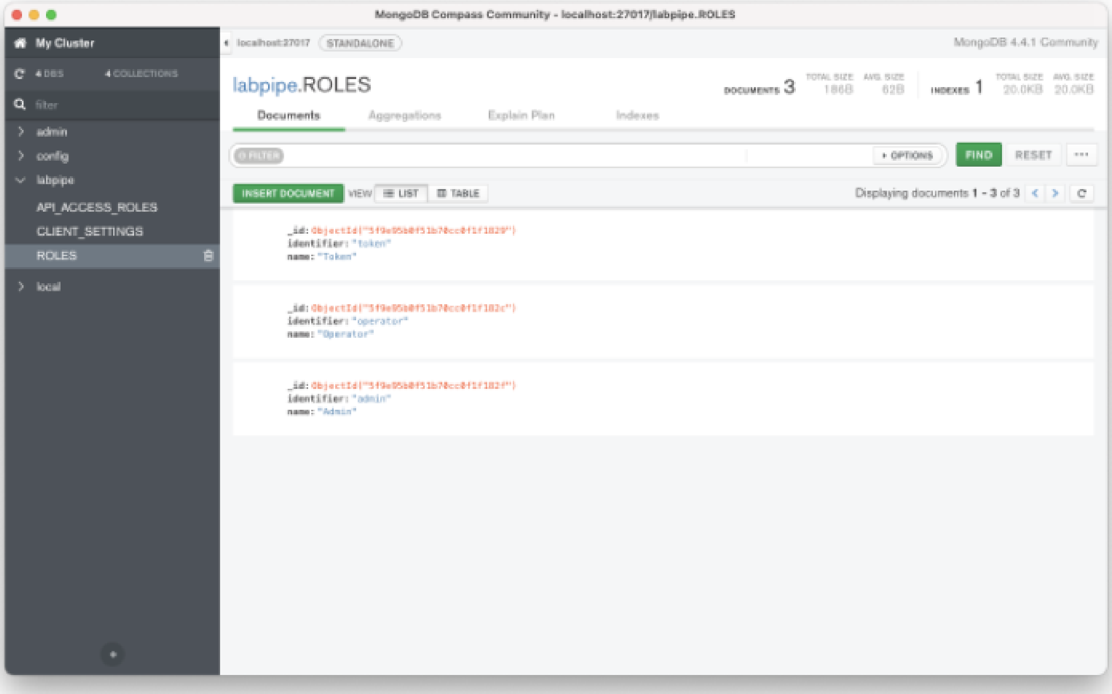

API_ACCESS_ROLES collection viewed using MongoDB Compass

Now you can start the LabPipe Server using:

```
java -jar labpipe-server-v{x.x.x}.jar run
```

Then you should see something like:

```
[main] INFO io.javalin.Javalin - Starting Javalin ...
[main] INFO io.javalin.Javalin - Listening on http://localhost:4567/
[main] INFO io.javalin.Javalin - Javalin started in 115ms \o/
[main] INFO uk.ac.le.ember.labpipe.server.cmdline.CommandLineUtil - Server running
```

Now your LabPipe Server is up and running. However, for it to work properly with LabPipe Client, we need to have values for `Location`, `Instrument`, `Operator`, `Study`, `FormTemplate` and `AccessToken`.

First, let's add an AccessToken record and an Operator record

`java -jar labpipe-server-v{x.x.x}.jar add operator --email test@labpipe.org --name LabPipe --show`

`java -jar labpipe-server-v{x.x.x}.jar add token`

You will see output like

```
[main] INFO uk.ac.le.ember.labpipe.server.services.ManageService - [Token] 455afbc6
[main] INFO uk.ac.le.ember.labpipe.server.services.ManageService - [Key] u7qxmS6CI8
[main] INFO uk.ac.le.ember.labpipe.server.services.ManageService - [Username] test@
[main] INFO uk.ac.le.ember.labpipe.server.services.ManageService - [Password] jQbYL
```

Make a note of these credentials, as passwords and token keys are stored with encryption in the database. We will use these credentials later with LabPipe Client.

```
java -jar labpipe-server-v{x.x.x}.jar add location --identifier s1 --name Site1
```

```
java -jar labpipe-server-v{x.x.x}.jar add location --identifier s2 --name Site2
java -jar labpipe-server-v{x.x.x}.jar add instrument --identifier i1 --name Instrument1 --
file-type=csv
java -jar labpipe-server-v{x.x.x}.jar add instrument --identifier i2 --name Instrument2 --
file-type=lst
```

Configure LabPipe Client

We assume this is the first time you have used LabPipe Client. When you start the software, you will be prompted for some mandatory settings:

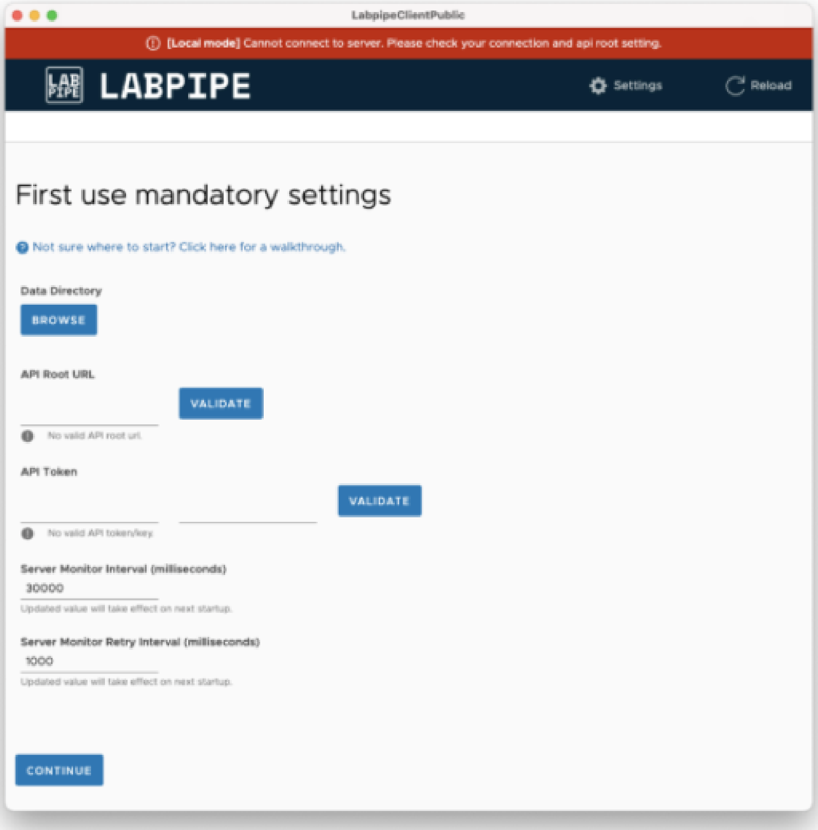

LabPipe Client first use mandatory settings

You should notice that the red banner now says ***Local mode***, which means the client is not connected to a LabPipe Server instance.

1. Select the directory to save metadata and data file locally
2. Enter LabPipe Server API root url ***http://localhost:4567***, then click ***VALIDATE***
3. Enter API token and key generated earlier, then click ***VALIDATE***
   Token: ***455afbc6-9d0b-4d68-a364-569ac456307f***
   Key: ***u7qxmS6Cl8JvHkt2***
4. Click ***CONTINUE*** and restart the client

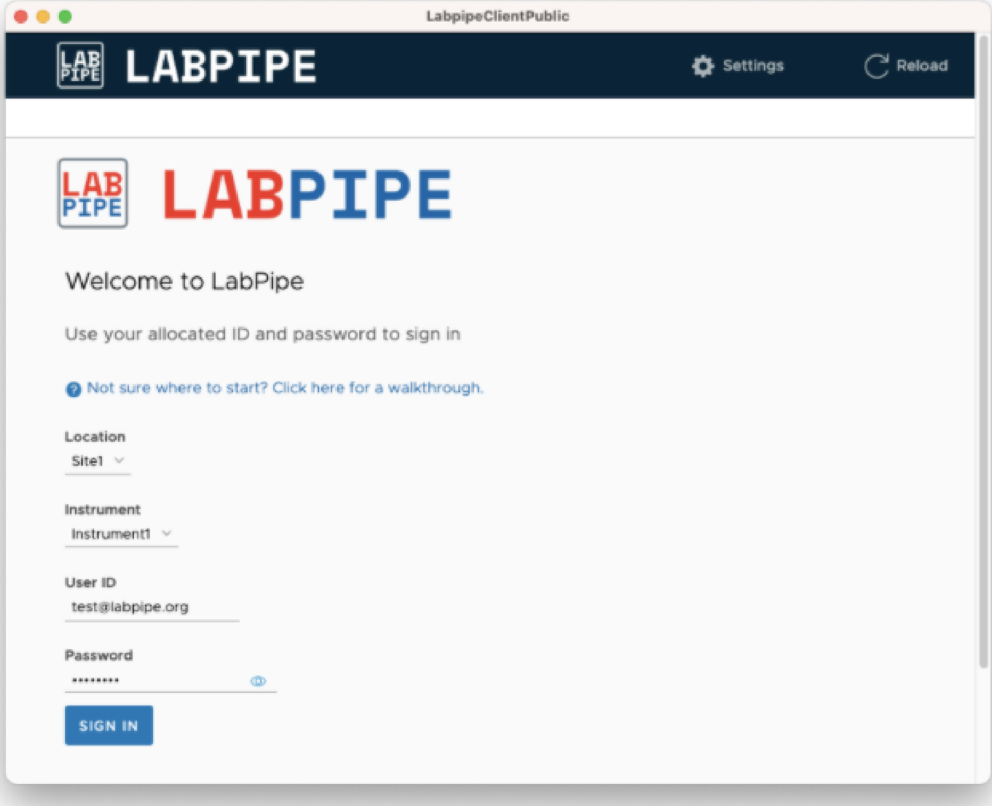

LabPipe login

Once restart completes, you can log in using the operator account added earlier. After logging in, you will notice that currently there is no study available to start. Now, let's add a study on the server.

Set up study and data collection form

Download an example study configuration [labpipe_example_study_1.json](), then run

```
java -jar labpipe-server-v{x.x.x}.jar --import study --source
path/to/labpipe_example_study_1.json
```

Download an example form template [labpipe_example_form_1.json](), then run

```
java -jar labpipe-server-v{x.x.x}.jar --import form --source
path/to/labpipe_example_form_1.json
```

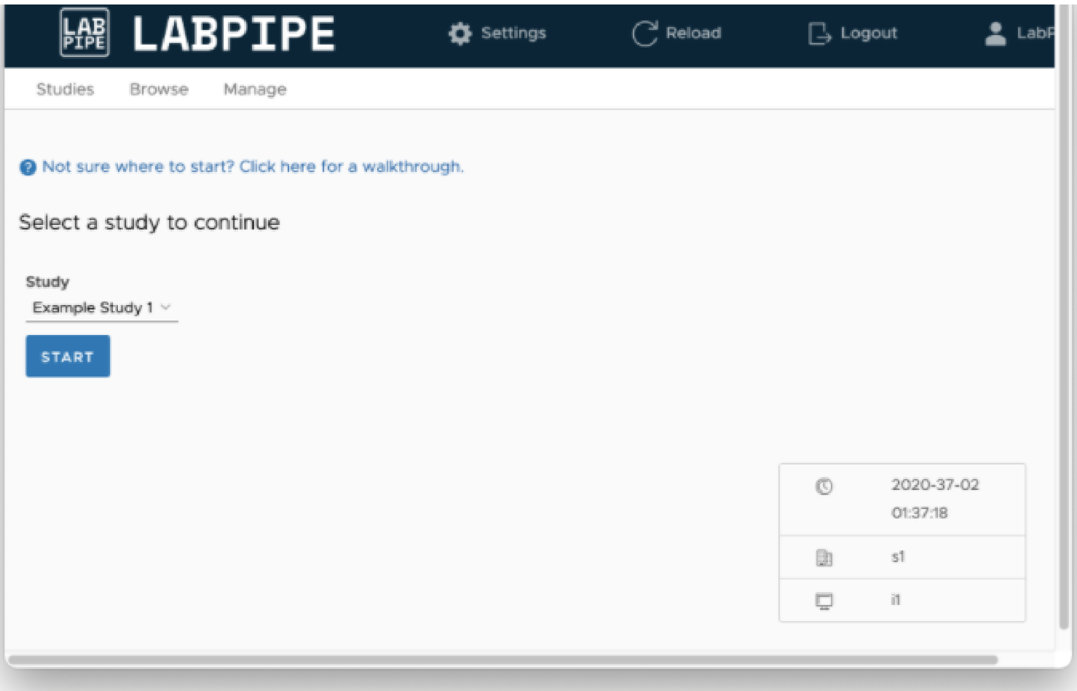

LabPipe Client select study

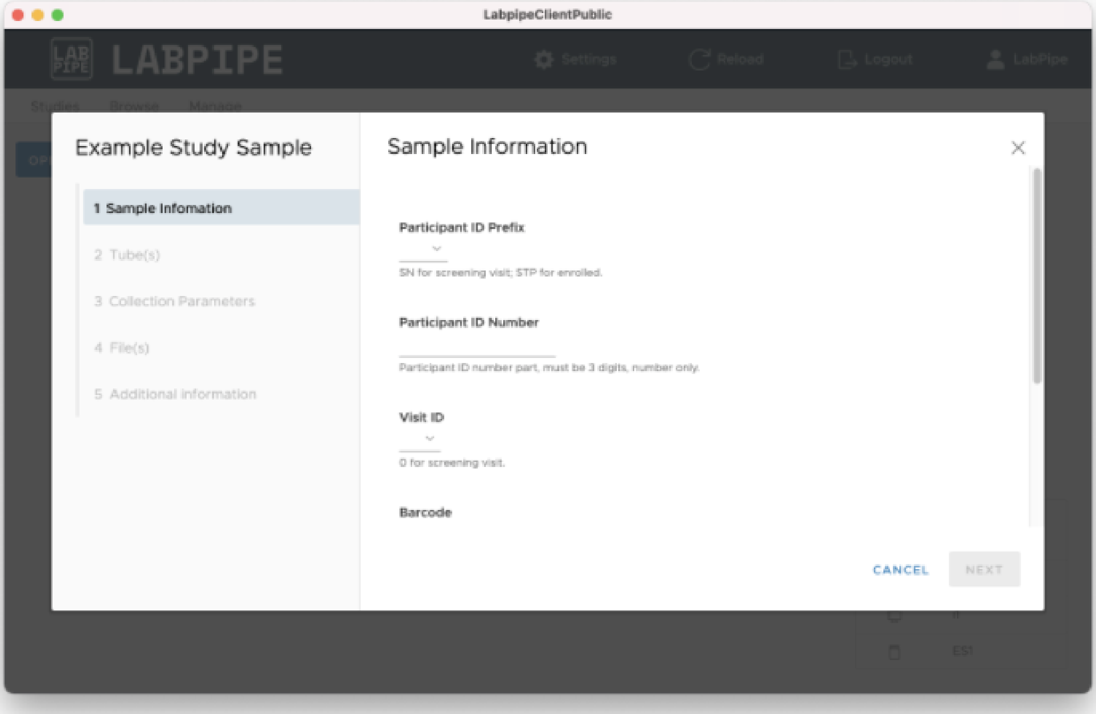

LabPipe Client data collection form

Now you can start collecting data using this example study sample form.

For additional documentation including example templates please go to www.labpipe.org
